# Maternal risk factors of COVID-19-affected pregnancies: A comparative analysis of symptomatic and asymptomatic COVID-19 from the Q-PRECIOUS registry

**DOI:** 10.5339/qmj.2022.52

**Published:** 2022-11-14

**Authors:** Fathima Minisha, Thomas Farrell, Salwa Abuyaqoub, Abubaker Abdel Rahim, Huda Ahmed, Mai Omer, Merlin Abraham, Franciscus Teunissen, Mahmoud Gassim, Q-PRECIOUS group

**Affiliations:** ^1^Department of Obstetrics and Gynecology, Women's Wellness and Research Centre, Hamad Medical Corporation, Doha, Qatar Email & ORCID ID: fathim999@gmail.com & ORCID- 0000-0001-6903-5445; ^2^Medical Research Centre, Hamad Medical Corporation, Doha, Qatar; ^3^Department of Pharmacy, Women's Wellness and Research Centre, Hamad Medical Corporation, Doha, Qatar

**Keywords:** Advanced maternal age, asthma in pregnancy, gestational diabetes, grand multiparity, high-risk pregnancy, Middle East, pre-existing diabetes, pregnancy-induced hypertension, pregnant women in Qatar, SARS-CoV-2

## Abstract

Background: The novel coronavirus disease 2019 (COVID-19) pandemic has had consequences on the pregnant population, as disease severity is associated with the quality of maternal health and pregnancy complications, increasing maternal and neonatal morbidity. Worldwide descriptive data help describe risk factors that could predict symptomatic and severe COVID-19 in pregnancy.

Objectives: To describe demographic features and risk factors of pregnant women with COVID-19 in Qatar and compare symptomatic versus asymptomatic disease.

Study design and methodology: Clinical characteristics and risk factors of pregnant women with COVID-19 in Qatar from March 2020 to March 2021 was retrospectively reviewed, comparing the cohort with the general pregnant population. Crude and adjusted odds ratios (aORs) were computed, comparing symptomatic versus asymptomatic infection.

Results: Of the 500 women, 347 reported at least one symptom at diagnosis (347/500; 69.4%). The majority fell in the 30–39 years age group (241/500; 48%), with more than half in the obese body mass index (BMI) category. The cohort was 66% (332/500) Qatari women, compared with the 26% expected in the population (26.4% vs 66.4% *p* < 0.001). Compared with the 2019 national statistics, the number of women was higher in the >40 years age group (5% vs 7.6%, *p* = 0.027) and grand multiparous group (5.4% vs 13.6%, *p* < 0.001). The symptom most commonly reported by the symptomatic group was cough (276/500; 55%), followed by fever, fatigue, and myalgia. In the adjusted analysis, the symptomatic group had 2.7 times higher odds of being asthmatic (OR = 2.67, 95% CI 1.1–6.7, *p* = 0.037). Women aged >40 years had 6.6 times higher odds of symptomatic disease (aOR = 6.6, 95% CI 1.08–39.73, *p* = 0.041). A history of contact with a patient with symptomatic COVID and earlier gestational age at diagnosis increased the odds (aOR = 2.06, 95% CI 1.2–3.54, *p* = 0.009; aOR = 0.73 95% CI 0.57–0.96; *p* = 0.017).

Conclusions: This study cohort included significantly more Qatari women, older women, grand multiparous women, a higher proportion with pre-existing and gestational diabetes, and higher BMI than national data. In addition, contact to a patient with symptomatic disease, history of asthma, older age, and earlier gestational age at diagnosis were significantly associated with symptomatic disease.

## Background

The novel coronavirus disease-2019 (COVID-19) caused by the severe acute respiratory syndrome coronavirus 2 (SARS-CoV-2) has infected more than 600 million people, of which 6.4 million have succumbed to the disease as of September 2022.^1^ This predominantly respiratory illness has a wide variation in presentation,^2,3^ with severe symptoms higher in patients with comorbidities and overall poor health.^4^ Identifying risk factors and implementing strict preventive measures have been the mainstay of disease control. The pregnant population are particularly vulnerable to the pandemic because of added concerns regarding the risks of available treatments and vaccines to the fetus.

Pregnant mothers respond to infections differently due to the combined effects of an altered immune response and fetal–placental immune system,^5^ leading to an unpredictable disease. The physiological respiratory changes during pregnancy^6^ increase their susceptibility to severe COVID-19. The increased requirement of access to healthcare in pregnancy also increases the exposure of these women to asymptomatic community cases. Higher rates of admissions to intensive care, severe respiratory and thromboembolic diseases, requirement for life support, and mortality have been persistent concerns for these women, as reported by the PAN-COVID UK registry and the Preg COV-19 systematic review.^7,8^ Severity appears to be associated with age, pre-existing medical conditions, obesity, ethnicity, and socioeconomic status.^9^

The Eastern Mediterranean global area accounted for 3.8% of the total COVID-19 cases in the world and 5.4% of the mortality as of September 2022.^1^ Specifically, the Middle East has a multi-ethnic younger population, with a high fertility rate.^10^ Here, women are more likely to be exposed to sick older family members and children under their care. However, no studies have described the characteristics of mothers with COVID-19 in this region, specifically from Qatar. This study compared risk factors of symptomatic with asymptomatic maternal COVID-19 using data from the Qatar pregnancy COVID outcome registry (Q-PRECIOUS).

## Methods

### Study design and setting

The Q-PRECIOUS is an active national perinatal registry, consisting of women diagnosed with COVID-19 during their pregnancies from March 2020. It includes all women receiving maternity care from public or private obstetric service providers within the State of Qatar. The registry was approved by the Medical Research Centre, Hamad Medical Corporation (HMC), Qatar (MRC-01-21-122) and by HMC Institutional Review Board, with a waiver of consent.

Data for this study were extracted from a retrospective chart review of cases from the first wave of the disease in Qatar (March 2020 to March 2021). The health card numbers of pregnant and postpartum patients were sourced directly from the Ministry of Public Health COVID-19 national records. Data from the first 500 eligible cases were collected from the Cerner Millennium® patient electronic health records. All data cleaning and analysis were conducted on-site by a team member not involved in the data collection.

### Study participants

COVID-19 was diagnosed following a positive reverse-transcriptase polymerase chain reaction (RT-PCR) naso-oropharyngeal swab test analyzed in the Central Department of Laboratory and Pathology, HMC. Women were screened due to clinical suspicion of COVID-19 as part of contact tracing or during screening for elective hospital admission. For inclusion in the registry, women had to be either confirmed to be pregnant by clinical examination and/or by urine or blood pregnancy test and/or a pelvic ultrasound revealing pregnancy, or to be within 6 weeks postpartum (the postpartum period was as defined by the World Health Organization and the period falling under the scope of maternal services offered by the hospital)

### Variable definitions and data source

**Figure 1.**
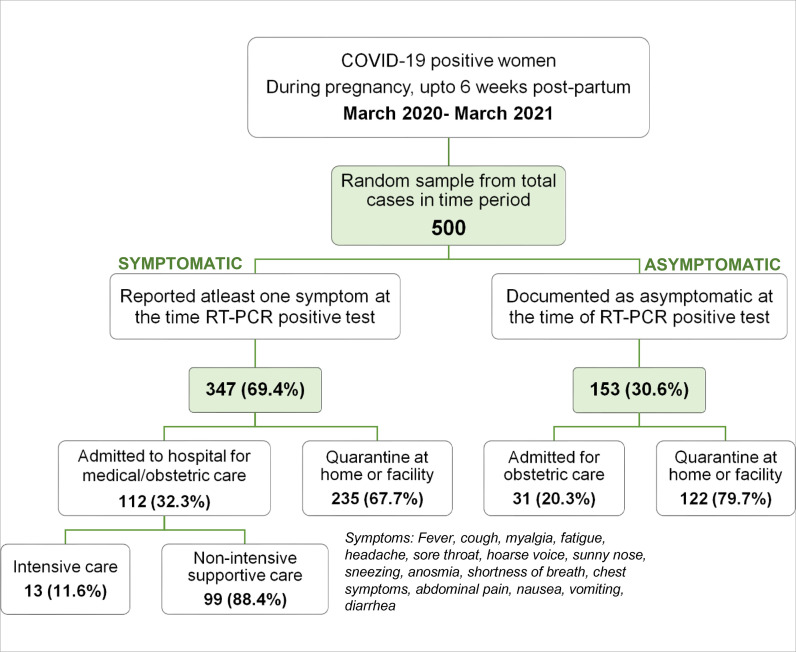
Study participant distribution; COVID-19- Novel coronavirus disease 2019; RT-PCR- Reverse transcription polymerase chain reaction

As part of the national COVID-19 prevention and control policies, any person testing positive was contacted by a health professional enquiring about symptoms. Women reporting any WHO-advised disease-related symptoms as detailed in Figure 1 ^11^ were assigned to the symptomatic group, and the remaining women composed the asymptomatic group.

Maternal age (in completed years), height and weight at diagnosis, and country of nationality were sourced directly from HMC medical records. The body mass index (BMI) was categorized according to the WHO BMI classification: underweight ( < 18 kg/m^2^), normal (18–24.99 kg/m^2^), overweight (25–29.99 kg/m^2^), and obese ( ≥ 30 kg/m^2^).^12^

Nationalities were categorized based on geographical regions: Qataris forming the baseline group, Arabs including women from the Middle East and North Africa, Asians including countries in the Indian subcontinent, and the far Southeast Asian countries. Pre-existing maternal medical conditions such as asthma, chronic hypertension, and diabetes mellitus (DM) were recorded as binary variables. Medical conditions with counts of < 5 were grouped together into the “other medical illness” category. They included cardiovascular diseases, hematological disorders, autoimmune conditions, renal diseases, gastrointestinal disorders, malignancies, etc.

The gestational age at diagnosis of COVID-19 was determined from the estimated date of delivery, based on either ultrasound or, when not available, last menstrual period (LMP). Gestation at time of COVID-19 infection was then categorized into four: first trimester (up to 13 completed weeks), second trimester (14–28 weeks), third trimester (29 weeks till the end of pregnancy), and postnatal (up to 42 days post-delivery). Parity was categorized as nulliparous (no prior children born after 24 weeks gestation), parous (previous 1–4 pregnancies beyond 24 weeks), and grand multiparous (>5 previous pregnancies beyond 24 weeks).^13^

Risk factors detected during pregnancy such as assisted reproduction, multiple gestations, hypothyroidism, gestational diabetes (GDM), gestational hypertension (including preeclampsia and eclampsia), and anemia were also collected as binary variables.

Hospital admission was either due to severe COVID-19 symptoms or obstetric indications. Similarly, the requirement for intensive care in symptomatic women was also collected.

The Planning and Statistic authority of Qatar releases annual vital statistics reports that include total live births in the year according to the maternal age at delivery, nationality, and parity. This publicly accessible 2019 Vital Statistics report was used as the baseline for the comparion of women in our study cohort.^14^

### Statistical analysis

Continuous variables were reported as mean and standard deviation and compared using Student's t-test. All categorical and ordinal variables were reported as frequency and percentage of the total in each comparison group using the Chi-square tests or Fisher's test as appropriate for analysis. For variables with more than two categories, the first category was considered the baseline. Logistic regression was used to determine the crude odds ratios (ORs) and 95% confidence intervals (CI) for each independent variable in the comparison of the symptomatic group with the asymptomatic group. In variables with ordered categories such as maternal age, BMI, parity, and gestational age at diagnosis, a test for trend was conducted to look for any increase or decrease in the ORs with increasing order of categories.

An exploratory logistic regression model was generated to determine the odds of symptomatic disease given each independent variable and adjusted for other variables. The variables in the model were clinically and historically relevant and included continuous variables (height in centimeters and weight in kilograms), ordinal variables (maternal age categories, gestational age at diagnosis of COVID-19, and nationality groups), dichotomous variables (history of asthma, chronic hypertension, history of flu vaccination in the past year, history of contact with symptomatic COVID, and gestational hypertension).

Variables with >20% missing data were not reported in the study. Those with >10% missing data were not considered for the regression analysis. A 95% CI not including one and/or a p-value of < 0.05 was considered significant. All analyses were performed using Stata IC version 16 (StataCorp LLC, College Station, TX, USA).^15^

## Results

### Descriptive results

Of the 500 women included, 347 reported at least one symptom at the time of diagnosis (69.4%, symptomatic group). More than a third (32%) of the symptomatic women required hospital admission, and 11% of those admitted required intensive care (Figure 1). The mean maternal age for the entire cohort was 30.9 (30.85 ± 5.76) years, with the majority belonging to the 30–39 years age group (48%). More than half of the women (54%) belong to the obese BMI category ( ≥ 30 kg/m^2^), with a mean BMI of 31 (30.68 ± 5.62). The nationals made up two-thirds (66%) of the cohort; only 3% of the cohort reported taking influenza vaccination in the past year (Table 1). A quarter of the women had a pre-existing medical illness, 8% were asthmatic, 7% were diabetic, and 1.5% had chronic hypertension.

Most of the women were diagnosed with COVID-19 in the second and third trimesters (Table 2). One-fifth of the women were in their first pregnancies compared with nearly 14% being grand multiparous. The most common pregnancy-related risk factor was GDM (41%), followed by thyroid disease and anaemia (17% and 15%, respectively). Only 4.5% of the cohort had gestational hypertension.

According to the National Statistics of 2019,^14^ only 26% of women giving birth in 2019 were Qatari, significantly contrasting with the 66% nationals in the study cohort (26.4% vs. 66.4% *p* < 0.001). In addition, the proportion of women with Arab and Asian nationalities were lesser than expected in the population (35% vs. 19.6%; 31.3% vs. 14.0%, respectively). Furthermore, the Q-PRECIOUS cohort had a greater number of women in the oldest age group (5% vs. 7.6%, *p* = 0.027) and more than double the proportion of grand multiparous women (5.4% vs. 13.6%, *p* < 0.001).

**Figure 2.**
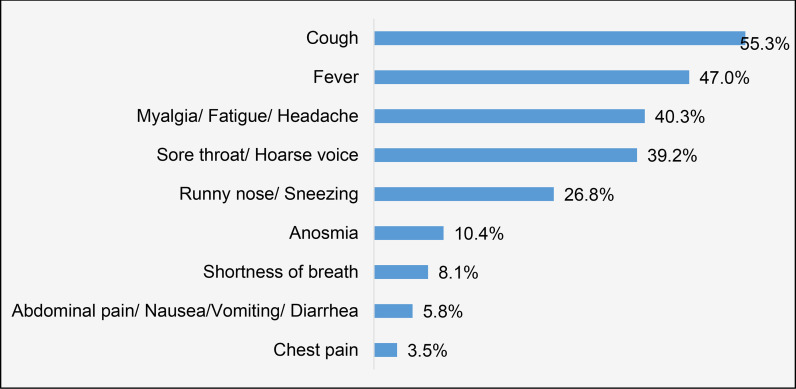
Percentage of symptomatic women presenting with various symptoms

The symptom most commonly reported by the symptomatic group was cough (55%), followed by fever (47%) and fatigue and myalgia (40%). Nearly 10% reported anosmia. However, < 10% of the women reported severe symptoms, including shortness of breath and chest pain, which was the least reported symptom (Figure 2).

### Crude analyses

The mean maternal age was comparable in the symptomatic and asymptomatic groups. However, a trend was noted in the maternal age categories, with the women in the oldest age group having 3.75 times higher odds of being symptomatic than women aged < 20 years (crude OR = 3.75, 95% CI 0.76–18.4). Women in the obese category were 58% more likely to be symptomatic than women in the normal BMI category (OR = 1.58, 95% CI 0.93–2.68). Both groups had an equal proportion of Qatari women (Table 1). Interestingly, women from the Arab region were 53% more likely to be symptomatic, contrasting with Asian women who were nearly 40% less likely to be so (OR = 1.53 vs. OR = 0.63). In addition, symptomatic women had a higher odds of being asthmatic (OR = 1.90, 95% CI 0.86–4.23), having contact with a patient with symptomatic COVID-19 (OR = 1.88, 95% CI 1.14–3.10), and being admitted to the hospital (OR = 1.88, 95% CI 1.19–2.95).

A trend was noted, i.e., symptomatic disease was related to the duration of pregnancy. Women had a higher odds of being asymptomatic as they advanced further in their pregnancy. More than half of the asymptomatic Asian women presented in the third trimester. In addition, symptomatic women were twice more likely to have gestational hypertension (OR = 2.05, 95% CI 0.68–6.06); the CI for these associations included 1 in the crude analysis (Table 2).

### Adjusted analyses

The logistic regression model determined the adjusted ORs (Table 3). After adjustments, symptomatic women had 2.7 times higher odds of being asthmatic (adjusted OR [aOR] = 2.67, 95% CI 1.1–6.7, *p* = 0.037). The trend observed in the maternal age category became more pronounced, with mothers aged>40 years having 6.6 times higher odds of symptomatic disease (aOR = 6.6, 95% CI 1.08–39.73, *p* = 0.041). A history of contact with a patient with symptomatic COVID doubled the odds of having symptomatic disease (aOR = 2.06, 95% CI 1.2–3.54, *p* = 0.009). These odds decreased by 27% when progressing through the trimesters of pregnancy (aOR = 0.73 95% CI 0.57–0.96; *p* = 0.017). The effect of nationality and history of chronic or gestational hypertension in predicting symptomatic disease decreased after adjusting for the other factors.

## Discussion

### Principle findings

This study describes the demographics and clinical characteristics of pregnant women in Qatar with symptomatic and asymptomatic COVID-19. This cohort had a higher proportion of Qatari, older, and grand multiparous women, obesity, and pre-existing DM and GDM than the general population. In the adjusted analysis, symptomatic women were at significantly higher odds of having asthma and being >40 years old and were more likely to be in the first or second trimester of pregnancy. None of the other risk factors was different between the groups.

### Clinical and research implications

In March 2021, Qatar had a population of 2.6 million of which 28% were women. Over the first wave, there were 181,000 COVID-19 cases in the country.^1^ Nearly 15% of those tested positive were women,^16^ with up to a thousand pregnant at diagnosis. This study looks at a representative random cohort of 500 pregnant women who had COVID-19 during this time, selecting across the 12 months proportional to the number of cases monthly. Thus, the results of this study can be generalized to all pregnant women in Qatar exposed to COVID-19 during this period.

In the study published in 2020 by Omrani et al., the 25–34 years age group was commonly infected.^17^ This is true of our cohort as well. However, relative to the national expectation, a higher proportion of older women were found in our cohort and were more likely to have symptomatic disease. This could be attributed to social circumstances where older women are more likely to be exposed to sick older relatives and children at home under their care.

The study by Omrani et al. and a 2021 study by Pathan et al. predicting COVID-19 in Qatar have reported that more people from the Southeast Asian region tested positive.^18^ By contrast, nearly two-thirds of the women in our cohort were Qatari. This proportion is significantly higher than the 26% expected proportion of Qatari mothers delivering annually in the population. To our knowledge, this is the first study looking at the nationalities of pregnant women with COVID-19. In addition, women from Asian regions were more likely to have asymptomatic disease. This was also observed clinically, as most of their diseases were picked up during routine admission screening.

The incidence of pre-existing DM and GDM in Qatar is 3%–4% and 24%, respectively.^19-21^ However, in this cohort, 7.4% had pre-existing DM, and 41% had new-onset GDM. This finding aligns with past publications that note the association between COVID and DM.^22^ GDM was found to be a risk factor for infection but not specifically for symptomatic disease. In Qatar, the incidence of gestational hypertension, including pre-eclampsia, is 4%, with 1% of pregnant women having chronic hypertension.^23^ Our cohort shows a similar prevalence, reinforcing that existing or newly developing hypertension in pregnancy did not increase the odds of COVID-19. A 2019 study reported that 58% of women delivering in Qatar had a pre-pregnancy BMI of >25 kg/m^2^.^24^ Moreover, 84% of Q-PRECIOUS women had a BMI of >25 kg/m^2^, supporting the evidence for obesity as a risk factor for COVID-19.

Our cohort has an almost equal distribution of cases between the three trimesters of pregnancy. Past publications have shown no variations in presentations among the trimesters, with the majority delivering at term.^25,26^ However, in this study, women diagnosed with COVID-19 in the third trimester were more likely to be asymptomatic. This could be attributed to more than half of asymptomatic Asian women being diagnosed when presenting in active labor in the third trimester.

Women exposed to symptomatic contacts were more likely to develop symptomatic disease. Although the prevalence of asthma in our cohort is as expected in the general population,^27^ they were more likely to experience symptomatic disease. The symptoms reported are similar to past publications,^7^ with fever and cough being the most common. However, we report fewer women (10% compared with 15% reported previously) with loss of taste and smell, possibly due to the initial unawareness of this symptom in the population. Our cohort had nearly 70% symptomatic women, and this agrees with the initial review of COVID-19 cases in Qatar, reporting that 69% of pregnant women were symptomatic.^17^ However, these figures are slightly less than the 77% symptomatic proportion reported in a large multicentric US CDC-based 2020 review.^28^ This could be attributed to the initial hesitancy in reporting symptoms because of uncertainties in the management and quarantine options available in the country.

### Strengths and limitations

The study is the first to report the characteristics and risk factors of pregnant women with symptomatic and asymptomatic COVID-19 in Qatar. It is unlikely to have a selection bias because the cohort was randomly selected and adequately represents the population. Data collection was performed by clinicians well versed in navigating the electronic medical records and interpreting the documentation. Further reviews were conducted to validate the data collected, ensuring data quality.

However, certain limitations should be highlighted because the data were collected from medical records. The grouping into symptomatic and asymptomatic cases was based on patient-reported symptoms, which can be subject to misclassification. Some women who were symptomatic might have been misclassified as asymptomatic due to the patient's unawareness or denial caused by the social stigma associated with the disease. However, this proportion is expected to be small, as the government officials have performed an exemplary job in risk assessment, triaging, and contact tracing.

Socioeconomic status and factors such as smoking and drug habits have been associated with COVID-19. However, the factors contributing to socioeconomic status such as education and household income were poorly reported in the medical records, with >50% missing data. Expectedly, these variables will be better extracted in the prospective phase of the registry. One of the potential concerns is the inflation of the false-positive rate at an alpha of 0.05 because many comparisons were made. However, we intend this study to be descriptive and emphasize reporting the point estimates and CIs rather than the *p*-value. The regression model is exploratory, and we intend to develop a prediction model for severe COVID-19 in pregnancy in the future.

## Conclusion

Nearly 70% of pregnant women with COVID-19 in Qatar reported at least one disease-related symptom. This study reports a higher proportion of Qatari women, older age, grand multiparous, obesity, and pre-existing DM and GDM than the expected national figures. In the adjusted analyses, exposure to a symptomatic case, history of asthma, older age, and earlier gestational age at diagnosis increased the odds of symptomatic disease. Further large-scale studies from the Q-PRECIOUS registry are needed to corroborate these findings.

### Conflicts of interest

The authors have no conflicts of interest to declare.

### Funding

This study did not require any funding.

### Author contributions

SW, TF, MG, and MA were involved in the setting up of the Q-PRECIOUS registry. FM, TF, SW, MG, AA, HA, and MO were involved in the design of the study and data collection. FT, FM, and MA were also involved in data processing and statistical analysis. All authors contributed towards drafting/revising the manuscript content. All authors read and approved the final version of the manuscript.

### Acknowledgements

We would like to acknowledge the members of the Q-PRECIOUS group responsible for setting up and successfully running the registry:

• Dr. Hilal Amin Tawfik Al-Rifai

• Dr. Shamsa Ahmad

• Dr. Huda Abdulla Hussain Saleh

• Dr. Lolwa Mohd. Abdulla I Alansari

• Dr. Mai Abdulla S A Al-Qubaisi

• Dr. Moza Sulaiman H Al Hail

• Dr. Muna A.Rahman S. Al.Maslamani

• Dr. Najat Ali Mohsen Khenyab

• Dr. Faten Altaher Mohd. Taha

• Dr. Zeena Saeed Bu Shurbak

• Dr. Mohammed A J Abukhattab

• Dr. Khalil Mohd. Khalil Salameh

• Dr. Anvar Paraparambil Vellamgot

• Dr. Mohd. Z.M. Abu Khalil

• Dr. Teresa Sandra Erice Rivero

• Mr. Shaban Fathy Kamel Mohammed

• Mr. Haseebur Rahman Khan Mohammed

• Ms. Haila Sowayed S. Johar

• Mr. Palli Valappila Abdul Rouf

• Mr. Binny Thomas

• Dr. Nader Izz Eddin Saleem Aldewik

• Dr. Naela S R Almallahi

• Dr. Ahmed Bahieeldin Mohamed Hassan Sweilim

• Dr. Amina Sayed Omar

• Dr. Anas Aljasem

• Dr. Devi Krishna Remadevi

• Dr. Ebtehag elfadil Ahmed

• Dr. Einass Isameldin A Wagealla

• Dr. Ekhlas Mohamed

• Dr. Emad Adel Alhajhasan

• Dr. Feras Moha. Kheir Qaddour

• Dr. Ghinwa Khodor Lawand

• Dr. Haifa Shaikh

• Dr. Hamda Ahmed Abdi

• Dr. Hind Mohamed Abdel Aal Mohamed

• Dr. Jaber Mohammed J A Alsulaiti

• Dr. Jis Thomas

• Dr. Komal Rafique

• Dr. Megha Misra

• Dr. Noor Saleh Ahmed Bawazir

• Dr. Nuda Elnagi Mousa Hago

• Dr. Riham Mosaad Ragab El Midany

• Dr. Sagda Abdelazim Hassabelrasoul Ahmed

• Dr. Sreenisha Sreenivasan Somini

• Dr. Wisam Ali Mohammad Al-Sheikh

• Dr. Yusra Mohamed.

### Affiliation

Women's Wellness and Research Centre, Hamad Medical Corporation, Doha, Qatar.

There is no conflicts of interest to declare for any members of the group.

## Figures and Tables

**Table 1:** Maternal demographics between symptomatic and asymptomatic COVID-19

Variables		Total N = 500		Symptomatic N = 347		Asymptomatic N = 153		Crude OR (95% CI)^$^

		n	%N	n	%N	n	%N	

Age (completed years)		30.85 ± 5.76		31.00 ± 5.79		30.51 ± 5.69		1.02 (0.98, 1.05)

Age in categories	< 20 years	8	1.6	4	1.2	4	2.6	1

	20–29 years	213	42.6	147	42.4	66	43.1	2.23 (0.54, 9.18)

	30–39 years	241	48.2	166	47.8	75	49	2.21 (0.54, 9.10)

	≥ 40 years	38	7.6 ↑	30	8.6	8	5.2	3.75 (0.76, 18.40)

Height in centimeters		158.63 ± 6.06		158.79 ± 6.04		158.28 ± 6.11		1.01 (0.98, 1.05)

Weight in kilograms		77.24 ± 15.08		78.05 ± 14.84		75.40 ± 15.51		1.01 (0.99, 1.02)

BMI (in kg/m^2^)		30.68 ± 5.62		30.93 ± 5.46		30.09 ± 5.95		1.03 (0.99, 1.06)

BMI categories	Normal	78	15.6	48	13.8	30	19.6	1

	Overweight	146	29.2 ↑	102	29.4	44	28.8	1.45 (0.81, 2.58)

	Obese	272	54.4 ↑	195	56.2	77	50.3	1.58 (0.93, 2.68)

	Underweight	4	0.8	2	0.6	2	1.3	0.63 (0.08, 4.68)

Nationality	Qatari	332	66.4 ↑	230	66.3	102	66.7	1

	Arab	98	19.6 ↓	76	21.9	22	14.4	1.53 (0.90, 2.60)

	Asian (SA & SEA)	70	14.0 ↓	41	11.8	29	19	0.63 (0.37, 1.06)

H/o flu vaccine in past year (Missing data = 36)		13	2.8	9	2.8	4	2.9	0.95 (0.29, 3.14)

H/o travel outside country		11	2.2	7	2	4	2.6	0.77 (0.22, 2.66)

H/o contact with a COVID-19 case		251	50.2	181	52.2	70	45.8	1.29 (0.88, 1.89)

H/o contact with a symptomatic COVID-19 case		114	22.8	90	25.9	24	15.7	1.88 (1.14, 3.10)

H/o asthma		41	8.2	33	9.5	8	5.2	1.90 (0.86, 4.23)

H/o chronic hypertension		7	1.4	6	1.7	1	0.7	2.67 (0.32, 22.41)

H/o diabetes mellitus		37	7.4 ↑	23	6.6	14	9.2	0.70 (0.35, 1.41)

H/o other major illness		42	8.4	31	8.9	11	7.2	1.27 (0.62, 2.60)

H/o chronic medication use		43	8.6	30	8.7	13	8.5	1.02 (0.52, 2.01)

Admission to hospital		143	28.6	112	32.3	31	20.2	1.88 (1.19, 2.95)


H/o, history of; OR, odds ratio; CI, confidence interval; BMI, body mass index; SA, South Asian; SEA, Southeast Asian; MD, missing data

↑ - higher than expected national figures; ↓ - lower than expected national figures

$- Baseline Asymptomatic

**Table 2:** Current pregnancy risk factors between symptomatic and asymptomatic COVID-19.

Variables		Total N = 500		Symptomatic N = 347		Asymptomatic N = 153		Crude OR (95% CI)

		n	%N	n	%N	n	%N	

Maternal parity^	Nulliparous	100	20	70	20.2	30	19.6	1

	1–4	332	66.4	230	66.3	102	66.6	0.97 (0.59, 1.57)

	Grand multipara	68	13.6 ↑	47	13.5	21	13.7	0.96 (0.49, 1.87)

Gestational age at diagnosis of COVID ^@^	1^st^ trimester	127	25.4	88	25.4	39	25.5	1

	2^nd^ trimester	176	35.2	133	38.3	43	28.1	1.37 (0.82, 2.28)

	3^rd^ trimester	177	35.4	115	33.1	62	40.5	0.82 (0.50, 1.34)

	Postnatal	20	4	11	3.2	9	5.9	0.54 (0.21, 1.41)

Multiple pregnancies		15	3	7	2	8	5.2	0.37 (0.13, 1.05)

Thyroid disease		85	17.2	61	17.8	24	15.9	1.15 (0.69, 1.93)

Gestational diabetes ^&^		202	41.1 ↑	140	41.1	62	41.1	1.00 (0.68, 1.48)

Gestational hypertension ^#^		22	4.5	18	5.3	4	2.7	2.05 (0.68, 6.16)

Anemia in current pregnancy		75	15.3	49	14.2	26	17.2	0.81 (0.48, 1.36)

H/o Recurrent miscarriage		30	6	20	5.8	10	6.6	0.87 (0.40, 1.92)


OR, odds ratio; CI, confidence interval; H/o, history of

^ births >24 gestational weeks or >500 g of birthweight

↑  higher than expected national figures; ↓  lower than expected national figures

@ First trimester, up to 13 completed weeks; second trimester, up to 28 completed weeks; third trimester, from 29 weeks till birth; postnatal, up to 42 days post-delivery.

& diabetes detected for the first time in this pregnancy; # hypertension developing during this pregnancy, including preeclampsia and eclampsia; thyroid, gestational diabetes, gestational hypertension, and anemia, 1% missing data

**Table 3:** Adjusted analysis for risk factors

Variables		Crude OR(95% CI)	Adjusted OR(95% CI)	P value

Maternal age in categories	< 20 years	1	1	

	20–29 years	2.23 (0.54, 9.18)	3.79 (.80,18.41)	0.099

	30–39 years	2.21 (0.54, 9.10)	4.43 (0.90, 21.76)	0.067

	≥ 40 years	3.75 (0.76, 18.40)	6.55 (1.08, 39.73)	0.041*

Nationality		0.88 (0.68, 1.14)	0.95 (0.72, 1.27)	0.741

H/o of asthma		1.90 (0.86, 4.23)	2.67 (1.06, 6.74)	0.037*

H/o contact with symptomatic COVID		1.88 (1.19, 2.95)	2.06 (1.20, 3.54)	0.009*

Gestational age at diagnosis		0.84 (0.67, 1.05)	0.73 (0.57, 0.95)	0.017*

Chronic hypertension		2.67 (0.32, 22.41)	1.45 (0.16, 13.63)	0.743

Gestational hypertension		2.05 (0.68, 6.16)	1.62 (0.51, 5.09)	0.413


Total N = 456; LR chi2(12) = 26.25; Prob > chi2 = 0.009;

The logistic regression model included maternal age, height, weight, nationality, gestational age, history of asthma, chronic hypertension, history of _flu vaccination in the past year, history of contact with symptomatic COVID, and current pregnancy hypertension.

OR, odds ratio; CI, confidence intervals; H/o, history of; N, total number of observations included in the model; LR, likelihood ratio; Prob>Chi2, probability more than the chi-square
